# Influence of institutional support and organisational culture on HIV and NCD integration

**DOI:** 10.4102/jphia.v16i1.1430

**Published:** 2025-10-29

**Authors:** David Njuguna, Caroline K. Kyalo, Eunice Mwangi, Daniel Mwai, Elvis Kirui, Wesley Rotich, Joy Mugambi, James Waweru, Nelson Muriu, Easter E. Olwanda

**Affiliations:** 1 Ministry of Health, Nairobi, Kenya; 2 Department of Health Systems Management, School of Medicine and Health Sciences, Kenya Methodist University, Nairobi, Kenya; 3 Department of Population Health, Aga Khan University-East Africa, Nairobi, Kenya; 4 School of Economics, University of Nairobi, Nairobi, Kenya; 5 The Executive Office of the President, Nairobi, Kenya; 6 Department of Data, Futures Health Economics and Metrics, Nairobi, Kenya; 7 Nakuru County Government, Nakuru, Kenya; 8 Nakuru Level 5 Hospital, Nakuru, Kenya; 9 Nyeri County Government, Nyeri, Kenya; 10 Department of Public Health and Monitoring and Evaluation, Futures Health Economics and Metrics, Nairobi, Kenya

**Keywords:** HIV, NCD, integration, institutional support, organisation culture, Kenya

## Abstract

**Background:**

The integration of services for human immunodeficiency virus (HIV) and non-communicable diseases (NCDs) has gained increasing attention in recent years because of the overlapping prevalence and shared risk factors between these health conditions. However, successful integration requires more than just the alignment of clinical practices. The role of institutional support and organisational culture in promoting effective integration remains an underexplored area.

**Aim:**

This study aims to fill this gap by examining how institutional structures and organisational values influence the integration of HIV and NCD care.

**Setting:**

The study setting was Nakuru County in Kenya.

**Methods:**

This study employed a qualitative research design to capture the nuanced experiences and perceptions of healthcare providers involved in HIV and NCD care integration. A total of 99 key informant interviews were conducted with healthcare providers in levels 2 to 5 facilities in Nakuru County. The interviews lasting 45 min – 60 min were conducted sequentially. This study adopted a thematic analysis using NVivo 12.

**Results:**

Institutional support, including an improved provider efficiency, support from top management, capacity building, availability of essential commodities, maximum use of facility space, and monitoring of outcomes, has been shown to enhance integration efforts. Additionally, a supportive organisational culture characterised by adaptivity, embracing innovative or new culture, staff empowerment to propose new strategies, teamwork and performance monitoring contributes to successful integration outcomes. These factors improve patient workflow, ensure continuity of care, reduce patient wait times and reduce stigma.

**Conclusion:**

The findings highlight the importance of leadership commitment, resource allocation, communication, collaboration, stigma reduction and patient-centredness in achieving successful integration outcomes.

**Contribution:**

This study contributes to the body of knowledge surrounding the integration of HIV and NCD services, providing valuable insights that can be applied in other contexts and settings aiming to enhance healthcare delivery and outcomes for individuals living with these conditions.

## Introduction

Co-morbidity between human immunodeficiency virus (HIV) and non-communicable diseases (NCDs) is common, as HIV infection can elevate the risk of developing NCDs. This increased risk is attributed to the activation of inflammatory markers and potential adverse effects of certain antiretroviral (ARV) medications used for HIV treatment.^1^ Traditionally, HIV and NCDs have been addressed separately, but there is a growing understanding of the need for integration.^2^ By integrating services, healthcare delivery becomes more efficient and comprehensive, resulting in improved health outcomes and cost-effective interventions.^3^ However, successful HIV and NCD integration goes beyond clinical and programmatic factors.

Institutional support includes investments in resources, creating an enabling work environment, and commitment of the top management to ensure efficient use of available resources.^4^ It is essential as it provides the necessary leadership, resources and guidance to drive the integration efforts. Continuous professional development is essential to stay abreast of advancements and prepare for emerging challenges. Evidence suggests that organisations should prioritise ongoing training initiatives to equip employees with the necessary skills to adapt to evolving job requirements and workplace changes.^5^ By providing continuous learning opportunities, institutions enable their workforce to acquire new knowledge and expertise, ensuring they can effectively respond to the dynamic demands of integrated services.^6^

Effective integration of HIV and NCD services within healthcare systems relies heavily on the underlying institutional support and organisational culture that shape implementation processes. These factors influence how new strategies are adopted, adapted, and sustained in practice. Building on this, a 2019 German study emphasised the importance of integrating patient access into student-centric medical education, proposing a digitalised approach to enhance learning and clinical practice. It proposed a four-step algorithm that utilised digitalised medical data to enhance patient access throughout curriculum development. The algorithm involved mapping learning objectives to relevant International Classification of Diseases (ICD)-11 codes, determining learning opportunities, assessing and selecting hospital departments with expertise in the objectives and assigning suitable patients for participation. The study underscored the need for trainees to possess adequate skills and advocated for realistic learning opportunities and assessments that integrated various aspects of medical practice in a team-based environment, fostering clinical reasoning and decision-making abilities.^7^ This highlights that organisational commitment and a culture conducive to innovation are critical components for successfully implementing integrated health services and improving patient outcomes.

Institutional support is also crucial for addressing the challenge of measuring and monitoring integrated care outcomes. To gain a better understanding of outcomes and patient satisfaction, it is important to consider domains such as coordination of care, continuity, patient-centredness, user-friendliness, community-based services, access and leadership.^8,9,10^ Additionally, the literature highlights the significance of incorporating domains that reflect the perspectives of managers, professionals and caregivers involved in integrated care. Institutional support plays a key role in implementing and evaluating integrated care initiatives to ensure improved outcomes and patient experiences.^11^ An empirical study utilising 278 survey responses indicates that institutional support, whether formal or informal, has a positive influence on the innovation outcomes.^12^

Institutional support is crucial for coordinating with external entities to promote integrated services and improve healthcare system performance. This collaboration facilitates the sharing of resources, expertise and information, enabling seamless coordination among different entities within the healthcare system.^13,14,15,16^ Institutional support ensures alignment in goals, policies and practices, leading to enhanced care coordination, efficient service delivery and better patient outcomes. By working closely with external entities, institutions can leverage collective knowledge and experience to drive innovation, implement evidence-based practices and address gaps in the healthcare system. This collaboration ultimately enhances the overall performance of integrated services and the healthcare system as a whole.^17^

In addition, the organisational culture within healthcare institutions plays a crucial role in integrating HIV and NCD services. A culture that encourages adaptivity and innovation empowers staff to explore new approaches and challenge traditional HIV and NCD care models.^18^ The process of integration involves developing new cultures that go beyond existing practices. A shared culture fosters learning and knowledge management, facilitating the exchange of new practices, policies and processes among healthcare workers. Being open to learning promotes knowledge exchange and supports the implementation of innovative approaches in care delivery.^19^

Openness to innovation is also crucial for integrating HIV and NCD services successfully. The innovation process begins with problem identification and analysis, serving as the foundation for developing solutions and making informed choices. Innovation processes lead to improved service quality and overall performance. A service integration strategy is an integral part of the innovation process and prioritising efficiency encourages adaptive and values-based services. However, organisations that deplete resources during innovation may become exhausted and fail to strengthen their innovation capacities.^20^

An outcome-oriented culture is also crucial for integrating HIV and NCDs. All partners involved in integration should monitor progress, engage in discussions and participate in planning and future research. By doing so, healthcare providers can make well-informed decisions about patient care and service delivery. To facilitate strategy implementation and enhance work performance, it is generally recommended to utilise a performance measurement system (PMS).^21^

Finally, a team-oriented culture is essential for integrating HIV and NCDs. Collaboration among healthcare professionals from different backgrounds promotes high-quality patient outcomes. In the past, healthcare providers worked in separate teams, each focusing on individualised care.^22^ However, there is now a shift towards comprehensive patient care, which requires hospitals to foster a culture of creating diverse and functional patient care teams.^23^ This can be achieved by inviting professionals from various disciplines to collaborate and bring their unique expertise to the table. These diverse teams can optimise their capabilities and skills, leading to improved integration, consistency and quality of treatment for patients with complex needs.^24^

The Harambee study was a cluster randomised trial conducted in Western Kenya that examined the impact, mechanisms and cost-effectiveness of integrating community-based HIV and NCDs care within microfinance groups on outcomes related to chronic disease management. The study demonstrated that it was feasible to recruit the required number of microfinance groups to ensure that the clinical trial was adequately powered. In addition, stakeholder feedback obtained through elicitation confirmed that the proposed intervention was generally acceptable and played a crucial role in identifying potential challenges before implementation.^25^ Building on this, understanding the broader context of institutional support and organisational culture is essential, as these factors significantly influence the success of integrating HIV and NCD care within community settings in regions such as Kenya, where the disease burden is notably high.

There is a knowledge gap in understanding how institutional support and organisational culture impact the integration of HIV and NCDs in Kenya: a region with a high disease burden. The level of resources, policies and infrastructure provided by healthcare institutions for integration, as well as the influence of organisational culture on collaboration among healthcare professionals, is not well-defined. This study aims to fill this gap by examining the role of institutional support and organisational culture in HIV and NCD integration in Kenya, providing valuable insights for evidence-based approaches and addressing the specific challenges faced in this high-burden region.

## Research methods and design

### Study setting

This study was conducted within the healthcare facilities in Nakuru County, Kenya, which are actively involved in providing care for individuals affected by HIV and NCDs. The selection of Nakuru County was based on the high prevalence of comorbidities between HIV and NCDs within the region. The facilities within Nakuru County share common observable characteristics, such as the provision of comprehensive care services, including diagnosis, treatment and management of both HIV and NCDs. These facilities serve as critical settings for investigating the integration of HIV and NCD care, given their focus on addressing the specific healthcare needs of individuals living with these conditions.

### Study design

This study employed qualitative research methods and utilised a cross-sectional design to gather data.

### Study participants

The participants were selected purposefully based on specific inclusion criteria, ensuring that the study obtained relevant insights into the areas of inquiry. The selection criteria included the willingness to provide informed consent and actively participate in the research, as well as possessing good knowledge or understanding of HIV and NCD integration. The study focused on healthcare providers who work in Level 2, 3 and 4 facilities in Nakuru County, specifically those involved in the integration of HIV and NCD care. Level 2 health dispensaries are operated by clinical officers and function similarly to health centres, although they lack inpatient facilities. Primarily located in urban areas, these dispensaries offer outpatient services, voluntary counselling and testing (VCT), tuberculosis treatment, laboratory diagnostics, well-baby clinics, antenatal and postnatal care, pharmacy services, counselling, curative treatments and referral letters to higher-level facilities. Level 3 health centres are small hospitals staffed by at least one doctor, clinical officers and nurses. They provide a wider range of services, including maternity inpatient wards, laboratory testing, dental care, HIV comprehensive clinics, diabetes and hypertension management, baby well clinics, antenatal and postnatal services and referrals to other facilities.

Level 4 county hospitals are larger, more comprehensive facilities managed by a medical director, often a doctor. They not only offer similar services to Level 3 hospitals but also include X-ray services. Typically, each county has one such hospital, with larger cities such as Nairobi hosting multiple facilities to serve the population.^26^ Table 1 provides further details on the participants involved in the study.

**TABLE 1:** Demographic characteristics.

Indicator	*n*	Total number of respondents	% of respondents
**Facility level**			
Level 5	1	-	-
Level 4	7	-	-
Level 3	12	-	-
Level 2	7	-	-
**Ownership**			
GOK	18	-	-
Private	7	-	-
FBO	2	-	-
**Respondents’ designation**			
Clinical Officers	39	15	55
Nurses	42	4	15
Medical officers	3	1	4
Laboratory technologist	3	1	4
Link desk personnel	3	1	4
Adherence counsellor	9	5	18
**Sex**			
Female	66	-	-
Male	33	-	-
**Years of professional experience**			
1–3 years	33	-	-
3–5 years	57	-	-
5–10 years	9	-	-

**Total**	**-**	**27**	**100**

GOK, Government of Kenya; FBO, Faith Based Organisation.

### Data collection

The primary method of data collection was through key informant interviews. These interviews were conducted with the selected healthcare providers. The interviews provided an opportunity for in-depth exploration of their perspectives, experiences and insights regarding the integration of HIV and NCD care. Detailed descriptive field notes were meticulously taken during the interviews, capturing a comprehensive account of the interactions between the interviewer and respondent, non-verbal communication cues, environmental factors and the interviewer’s reflections on the interview content. Each respondent participated in a single interview session. Following each interview, the transcripts were carefully reviewed to verify the accuracy of the recordings and the completeness of the data. The interviews were transcribed verbatim in Swahili, and the transcriptions were cross-checked multiple times to ensure the accuracy and quality of the data before proceeding with the data analysis.

### Data analysis

A thematic analysis approach was employed. The English transcripts were thoroughly reviewed multiple times to develop a deep understanding of the raw data. Open coding was employed to identify and assign codes to the role of institutional support and organisational culture in HIV and NCD integration. Subsequently, axial coding was conducted to establish relationships between codes and to categorise them based on shared concepts, dimensions and properties. Finally, selective coding was performed to focus on the core concepts identified from the data. In the initial phase of analysis, researcher 1. independently conducted the coding. This was followed by collaborative discussions between researcher 1 and researcher 2 where emerging codes were compared, and a consensus was reached on a final coding framework. The coded data were then analysed using NVivo 12 software (see Table 2).

**TABLE 2:** Selective coding table.

Serial number	Selective codes	Axial codes	Open codes
1	Institutional support	Improved provider efficiency	Support from top management
			Capacity building
			Availability of essential commodities
			Maximum use of facility space
			Monitoring of outcomes
			Collaboration with external partners
		Improved patient orkflow	Continuity of care
			Reduced patient wait times
			Reduced stigma
2	Organisation Culture	Adaptivity	Embracing innovative or new culture
			Staff can propose new strategies
			Teamwork
			Performance monitoring

### Trustworthiness

To ensure trustworthiness in this study, various techniques were employed, following established criteria for credibility, dependability, confirmability and transferability. Two researchers engaged in debriefing sessions after the interviews and developed interview summaries, promoting a deeper understanding of the collected data. Independent coding of initial interview data by two researchers was conducted, and a coding framework was established through consensus, leading to the identification of emergent themes and categories. The research team held regular meetings to review and discuss subthemes, themes and categories, addressing any disagreements until a consensus was reached. Confirmability was ensured by supporting the results with direct quotes from participants, thus maintaining transparency and traceability. To establish dependability and replicability, a clear and systematic procedure for data collection and analysis was outlined. Finally, transferability was addressed by purposefully sampling participants with diverse characteristics, allowing for potential generalisation of findings to similar contexts.

### Ethical considerations

Ethical clearance to conduct this study was obtained from the Kenya Methodist University, Scientific Ethics and Review Committee (SERC)(KEMU/SERC/HSM/27/2022), as well as the National Council of Science, Technology and Innovation (NACOSTI) NACOSTI/P/23/22899. Additionally, approval was obtained from the County Department of Health (ref. NCG/CDPH/RES/VOL.1/2023/667) and the relevant health facilities. Prior to participating in the study, informed consent was obtained from all respondents, ensuring their willingness to provide feedback. The confidentiality of collected data was strictly maintained, with only the researcher having access to the information. This study adhered to rigorous ethical standards for human research participants throughout all stages of implementation, as outlined by Mugenda (2008).

## Results

The findings of this study reveal that institutional support plays a crucial role in facilitating the successful integration of HIV and NCD services.

### Institutional support

The findings reveal a mixed response regarding the level of support from top management for integrating HIV and NCD health services within the health facility. Among the total respondents, 15 out of 27 acknowledged receiving institutional support, while 12 out of 27 expressed a lack of support from the top management. Among those who received support, a prominent driving factor was the shortage of staff and the need to optimise resources. A healthcare provider explained this by saying:

‘Without the unwavering support and commitment from top management, our efforts to integrate HIV and NCD services would have been impossible. They have provided us with the necessary resources, guidance, and strategic direction to make this integration a reality. Their support has allowed us to allocate dedicated staff, secure essential commodities, and optimize facility space for efficient service delivery. They have also fostered collaboration with external partners, enabling us to tap into additional expertise and resources. Their leadership has created an environment where innovation is encouraged, empowering our staff to propose new strategies and approaches. This top management support has truly been the driving force behind our successful integration, ultimately benefiting our patients and improving their overall health outcomes.’ (Participant 1, Clinical officer)

Furthermore, institutional support for HIV and NCD integration is also reflected in the provision of infrastructure that facilitates comprehensive care. As one healthcare provider acknowledged:

‘Our facility has been equipped with specialized examination rooms and equipment that cater to the needs of patients with both HIV and NCDs. This infrastructure allows us to provide integrated services seamlessly, ensuring that patients receive comprehensive and holistic care under one roof. The availability of dedicated spaces for counseling, medication dispensing, and follow-up visits has streamlined our workflow and improved the overall patient experience. The investment in this infrastructure demonstrates the commitment of our institution to support the integration of HIV and NCD services, ultimately enhancing the quality of care we deliver to our patients.’ (Participant 2, Clinical officer)

This quote emphasises the importance of having a supportive institutional framework in place that recognises the need for integrated care.

Another challenge in institutional support was the difficulty in identifying the healthcare needs of patients with NCDs within the existing electronic medical record (EMR) system. This limitation prevented health workers from accurately accounting for the number of HIV patients who also have underlying chronic illnesses:

‘The current EMR system doesn’t allow easy identification of patient’s NCDs needs, therefore a health worker can’t account for the number of HIV patients who also have an underlying chronic illness.’ (Participant 3, Clinical officer)

The recommendations put forth to gain institutional support for the effective implementation of HIV and NCD service integration encompass a range of strategies. These include leveraging digitalisation, augmenting staff, and infrastructure, providing comprehensive training, sharing successful models and introducing integration models, proper planning and resource allocation, as well as securing donor presence and financial support. Through the implementation of these proposals, healthcare systems can significantly enhance the coordination and delivery of care for individuals living with HIV and NCDs:

‘I believe it is crucial that we prioritize movement towards adequate staffing, better infrastructure, training, and provision of adequate space. These are essential elements for achieving a streamlined integration of HIV and non-communicable disease (NCD) services. Without these improvements, it will remain challenging to effectively address the healthcare needs of patients living with both HIV and underlying chronic illnesses.’ (Participant 1, Nursing officer)

The respondents cited significant benefits to the patients resulting from the integration of HIV and NCD services, including improved continuity of care, reduced time spent in healthcare facilities and increased patient comfort with familiar healthcare providers within a specific department, which facilitates better communication and adherence to clinics. In addition, the integration helps to mitigate feelings of isolation among Comprehensive Care Clinics (CCC) patients, thereby reducing stigma. Furthermore, it enables the pooling of resources, enhancing efficiency and effectiveness in healthcare delivery:

‘By integrating HIV and NCD services, we have witnessed remarkable improvements in patient care. Patients now experience reduced time spent in healthcare facilities, there is no stigmatization, and we are able to pool resources effectively. Most importantly, their needs are addressed comprehensively, ensuring a holistic approach to their well-being.’ (Participant 2, Nursing officer)

The integration of HIV and NCD services also yields substantial benefits in terms of facility utilisation, as highlighted by the respondents. These benefits encompass cost savings, optimal utilisation of space and personnel, and the establishment of a systematic allocation of departments, promoting a smooth flow of patients and reducing confusion. From a health systems perspective, such integration has proven to be an effective strategy for enhancing overall operational efficiency and resource allocation within healthcare facilities:

‘The integration of HIV and NCD services has demonstrated remarkable efficiency in resource utilization. It has allowed facilities to optimize their resources, resulting in cost savings and requiring less infrastructure and personnel. This streamlined approach not only enhances the effectiveness of healthcare delivery but also ensures that limited resources are utilized wisely, ultimately benefiting both patients and healthcare systems.’ (Participant 3, Nursing officer)

In addition, integration contributes to increased job satisfaction among healthcare workers, as they can deliver more comprehensive and patient-centred care, ultimately leading to improved outcomes and a sense of fulfilment in their roles:

‘With the integration of HIV and NCD services, we have witnessed a significant improvement in the continuity of care. It has become much easier to comprehend a patient’s complete medical history and track their current progress. This comprehensive understanding allows us to provide more personalized and effective healthcare, ensuring that patients receive the seamless and continuous care they need for their overall well-being.’ (Participant 4, Nursing officer)

### Organisational culture

Overall, a significant number of facilities have taken initiatives to enhance the skills and knowledge of healthcare providers in managing the integrated care of HIV and NCDs. The majority (*n* = 17 out of 27) indicated that their facility had conducted training and held workshops to build capacity for care providers in handling HIV and NCD services. Ten facilities responded negatively, stating that they had not organised any such training sessions. Furthermore, the participants recognised the significance of providing comprehensive training to employees, enabling them to acquire the necessary skills to effectively implement an integrated care model. This training would equip them with the ability to proficiently manage the complex needs of HIV patients with multiple diagnoses, ensuring appropriate and tailored care, enhancing job efficiency, fostering staff confidence, and promoting competency and efficiency:

‘Training staff on HIV NCD integration guarantees effective management of patients by keeping staff members updated on the current changing health dynamics, ultimately enhancing the quality of care provided.’ (Participant 4, Clinical officer)

The majority of respondents recognised that to optimise service provision and ensure ongoing enhancement in service delivery, facilities implemented various strategies for monitoring performance outcomes. These included regular continuous quality improvement (CQI) meetings, comprehensive weekly and monthly reports, monthly and quarterly meetings, as well as continuous medical education initiatives. By utilising these mechanisms, facilities were able to maintain a proactive approach to quality improvement and keep abreast of emerging trends and best practices in healthcare delivery.

Finally, several external stakeholders and institutions collaborated closely with these facilities to provide valuable support for the integrated care model, with a particular focus on HIV and NCD services. Notable partners included Liverpool Voluntary Counselling and Training Centre (VCT), Marie Stopes, United States Agency for International Development (USAID) TUJENGE JAMII, through the Ministry of Health (MOH), Kenya Medical Supplies Authority (KEMSA), Mission for Essential Drugs and Supplies (MEDS), The United States President’s Emergency Plan for AIDS Relief (PEPFAR), Tuberculosis Antibiotic Resistance Catalog (TB-ARC), Centre for Health Solutions Kenya Medical Supplies Authority, Mission for Essential Drugs and Supplies, Centre for Health Solutions and the Government of Kenya. These collaborative efforts fostered a comprehensive and holistic approach to healthcare, ensuring the availability of resources, expertise and funding necessary to deliver high-quality, integrated care to individuals in need. These institutions predominantly extended support to health facilities in various critical areas, including human resources and financial assistance. In addition, they provided valuable contributions such as family planning services, provision of ARV medications, as well as essential commodities and equipment necessary for effective healthcare delivery. Their multifaceted support played a vital role in strengthening the capacity and resources of the health facilities, enabling them to provide comprehensive and high-quality care to their patients.

In all facilities, the staff exhibited a remarkable openness to change and readily embraced new approaches to streamline operations. The staff members had the freedom to propose and implement innovative methods of accomplishing tasks, fostering a collaborative team environment. Furthermore, each facility diligently set goals for its staff, which were conscientiously measured through regular performance evaluations conducted on a monthly, quarterly and annual basis:

‘At our facility, we set goals for our facility staff and evaluate these goals through the diligent efforts of our management team. We are committed to fostering growth and improvement and provide feedback during our regular monthly, quarterly, and annual meetings. This feedback serves as a catalyst for continuous development, ensuring that our staff members are well-supported and aligned with our organizational objectives.’ (Participant 5, Nursing officer)

## Discussion

This study highlights the impact of institutional support and organisational culture on the integration of HIV and NCD in Nakuru County, Kenya. The findings delve into the intricate relationship between these factors and shed light on their significance in promoting successful HIV and NCD integration efforts. Improved provider efficiency is one aspect of institutional support, and it is achieved through various factors. Support from top management is essential as it provides the necessary leadership, resources, and guidance to drive the integration efforts. Capacity building ensures that healthcare providers have the knowledge and skills required to deliver integrated care effectively. The availability of essential commodities and maximum use of facility space are crucial for ensuring that healthcare facilities are well-equipped and utilised optimally to provide comprehensive services. Improved monitoring of outcomes allows institutions to track their progress, identify areas for improvement, and make data-driven decisions to enhance the quality of care. Collaboration with external partners, such as non-governmental organisations (NGOs) or community organisations, strengthens the support network and brings additional expertise and resources to the integration process.

Institutional support plays a crucial role in enabling teams to identify and address specific needs in healthcare through interprofessional collaboration.^27^ It facilitates professionals and services in working together as a network to achieve health policies, as highlighted by the findings of this study. The surveyed facilities are commended for providing continuous improvement training on emerging health issues. This investment demonstrates their commitment to excellence and adaptability in the face of evolving healthcare challenges. It fosters a culture of lifelong learning and professional growth among staff members, enabling them to deliver high-quality healthcare services. The full support provided by organisations for staff workshops, conference participation, and seminars is also a valuable finding as it allows healthcare professionals to engage with peers, share knowledge and learn from experts. The study corroborates the findings of Dastagir et al., which showed that an intensive training programme led by physicians had a significant impact on experienced users of Electronic Health Records (EHR).^28^ By encouraging staff members to participate in these events, healthcare facilities foster a dynamic learning environment and empower their employees to stay at the forefront of advancements in their respective fields.^29^

Conceptualising collaborations as one component in a complex health system may help us promote HIV and NCD integration.^30^ The close collaboration between healthcare facilities and learning institutions is a strategic approach to ensure that educational programmes align with the needs of the health sector as it might help organisations combine their skills and resources to better meet community needs. The study highlights a potential opportunity for knowledge dissemination and collaboration through fully sponsored degree programmes, as very few faculty staff members have received such support.^15^ Engaging in teaching roles allows healthcare professionals to share expertise, contribute to education and enhance their own professional development. Organisations should encourage and facilitate staff involvement in teaching to capitalise on this potential.

Monitoring outcomes is another crucial finding that points to institutional support in maintaining and improving the quality of healthcare services in the surveyed health facilities. These health facilities monitor outcomes by conducting customer satisfaction surveys on a frequent basis to identify patterns and trends, pinpoint specific areas for improvement and make informed decisions. In line with this study, Chinomona and Moloi^31^ conducted research on the impact of institutional support on the commitment, satisfaction and performance of teachers in South Africa. Their findings revealed that institutional support has a positive effect on institutional commitment, job satisfaction and employee performance.^31^ Additionally, client ratings and recommendations are critical for accountability and improvement, helping to shape service delivery in real-time.^32^ Moreover, involving staff in problem identification is crucial for health facilities to utilise the collective knowledge and experience of their workforce. Empowering staff to voice concerns and suggestions allows facilities to tap into their expertise and gain deeper insights into factors affecting HIV and NCD service delivery. This collaborative approach fosters accountability, ownership, commitment and motivation among staff members. Regular outcome monitoring and continuous staff involvement in problem-solving ensure that the facility remains responsive to patient needs and maintains a high standard of care. There was, however, a need to improve on monitoring outcomes in Nakuru County, because this would lead to improved performance of the health system. This is largely driven by the lack of training that was mentioned by respondents in this study.

In addition to institutional support, the study also emphasises the pivotal role of organisational culture in facilitating successful integration. A meaningful, sustainable and purposeful organisational culture is essential, characterised by a clear sense of direction. It is crucial for staff members to establish a profound connection and comprehensive understanding of the values that form the bedrock of the organisational culture.^33^ By analysing the existing culture, our study identifies both enablers and barriers to integration efforts. It highlights the importance of fostering a culture of collaboration, innovation and inclusivity, where healthcare professionals work cohesively across disciplines and departments to deliver comprehensive care for individuals with HIV and NCDs. This finding is consistent with that of Munir, Samina and Stephen Kay, who conducted a study on organisational culture and systems integration in healthcare, specifically in intensive care settings. They found that actual usefulness and organisational culture have a direct impact on Clinical Information Systems (CIS) integration.^34^

As a cross-sectional exploratory study, the findings are limited to the specific context and time frame of the research. The use of purposive sampling may introduce selection bias, and therefore the findings may not be generalisable to the broader population. However, the study design allows for in-depth exploration and provides valuable insights into the perspectives of the selected healthcare providers regarding institutional support and organisational culture in HIV and NCD integration.

In conclusion, the successful integration of HIV and NCD services relies on a strong organisational culture and institutional support. A culture that promotes collaboration, patient-centred care and innovation, along with institutional commitment, leadership and resource allocation, are critical elements. By fostering such an environment, healthcare systems can address the complex health needs of individuals living with both HIV and NCDs, leading to improved health outcomes and overall well-being.

The findings of this study have significant implications for policymakers, healthcare administrators and practitioners involved in the integration of HIV and NCD services in Nakuru County. By recognising the importance of institutional support and organisational culture, stakeholders can develop targeted strategies to address gaps, allocate resources effectively and create an enabling environment for successful integration. Ultimately, this research contributes to the body of knowledge surrounding the integration of HIV and NCD services, providing valuable insights that can be applied in other contexts and settings aiming to enhance healthcare delivery and outcomes for individuals living with these conditions.
